# Running economy: measurement, norms, and determining factors

**DOI:** 10.1186/s40798-015-0007-y

**Published:** 2015-03-27

**Authors:** Kyle R Barnes, Andrew E Kilding

**Affiliations:** 1 Sports Performance Research Institute New Zealand, AUT University, Auckland, New Zealand; 2 Department of Movement Science, Grand Valley State University, Allendale, Michigan USA

## Abstract

Running economy (RE) is considered an important physiological measure for endurance athletes, especially distance runners. This review considers 1) how RE is defined and measured and 2) physiological and biomechanical factors that determine or influence RE. It is difficult to accurately ascertain what is good, average, and poor RE between athletes and studies due to variation in protocols, gas-analysis systems, and data averaging techniques. However, representative RE values for different caliber of male and female runners can be identified from existing literature with mostly clear delineations in oxygen uptake across a range of speeds in moderately and highly trained and elite runners. Despite being simple to measure and acceptably reliable, it is evident that RE is a complex, multifactorial concept that reflects the integrated composite of a variety of metabolic, cardiorespiratory, biomechanical and neuromuscular characteristics that are unique to the individual. Metabolic efficiency refers to the utilization of available energy to facilitate optimal performance, whereas cardiopulmonary efficiency refers to a reduced work output for the processes related to oxygen transport and utilization. Biomechanical and neuromuscular characteristics refer to the interaction between the neural and musculoskeletal systems and their ability to convert power output into translocation and therefore performance. Of the numerous metabolic, cardiopulmonary, biomechanical and neuromuscular characteristics contributing to RE, many of these are able to adapt through training or other interventions resulting in improved RE.

## Key points

- Running economy is a complex, multifactorial concept that represents the sum of various metabolic, cardiorespiratory, biomechanical and neuromuscular characteristics during submaximal running.
- Many of the determining factors of running economy are able to adapt through training or other interventions, however an economical change in one athlete may be uneconomical in another athlete because of differences in other physiological or biomechanical characteristics.
- Representative running economy values for different caliber of runners running at various speeds are presented.

## Introduction

**Figure 1:**
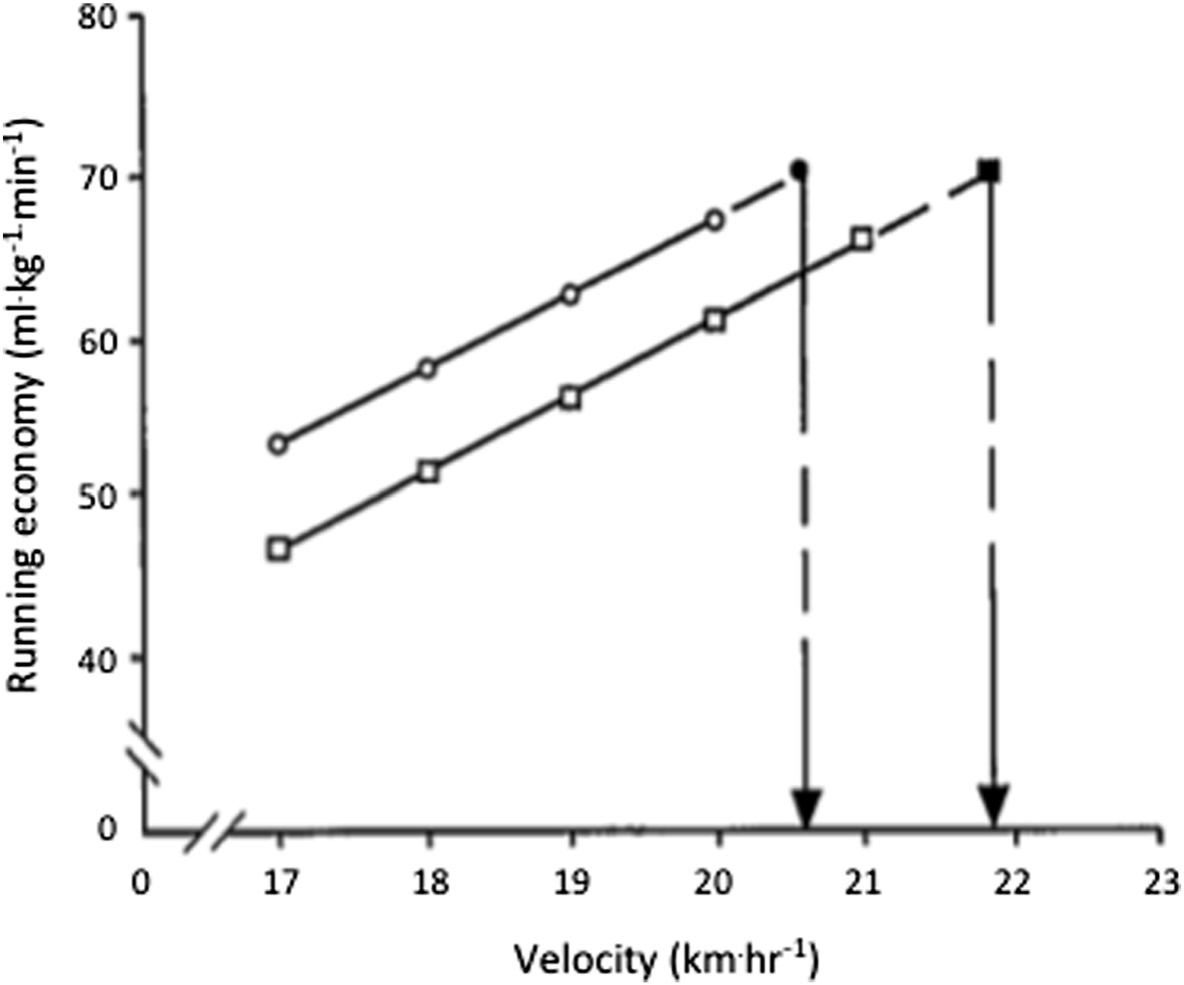
**Running economy profiles of two runners of equal VO**_**2**_ **max.**

The steady-state oxygen consumption (VO_2_) at a given running velocity, which is often referred to as running economy (RE) [1-3], reflects the energy demand of running at a constant submaximal speed. Runners with good economy use less oxygen than runners with poor economy at the same steady-state speed (Figure 1) [4]. It has been reported that RE can vary by as much as 30% among trained runners with similar VO_2_max [2]. Running economy has also been shown to be a useful predictor of endurance running performance [1,2,5-8] especially in athletes who are homogenous with respect to VO_2_max (Figure 1) [1,6,9].

**Figure 2:**
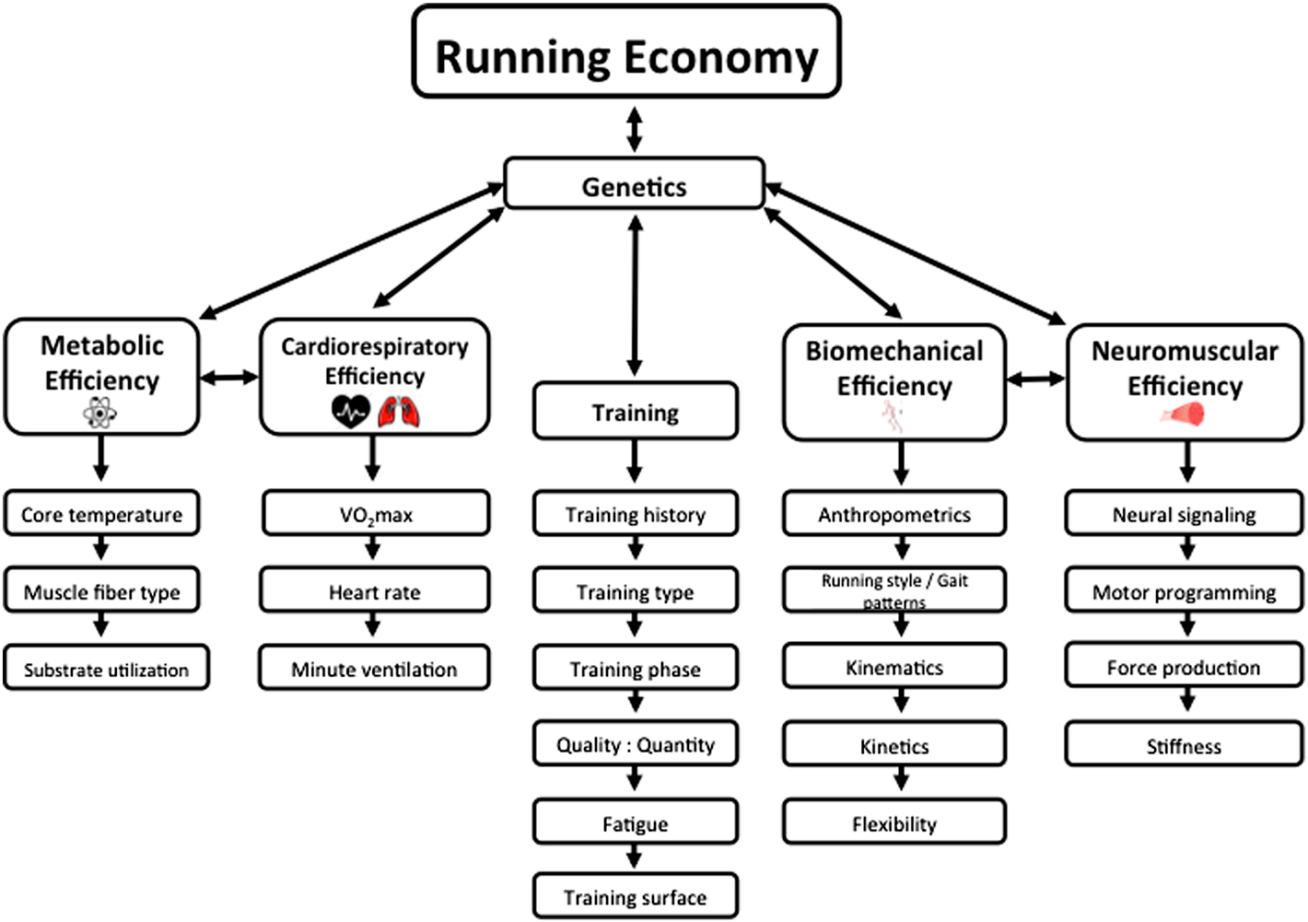
**Factors affecting running economy.**

While the measurement of RE is often perceived as a simple concept, it is actually a multifactorial measure which reflects the combined functioning of the metabolic, cardiopulmonary, biomechanical and neuromuscular systems (Figure 2) [2,3,5,10]. Metabolic efficiency refers to the utilization of available energy to facilitate optimal performance [2,3], whereas cardiopulmonary efficiency refers to a reduced work output for the processes related to oxygen transport and utilization. Lastly, neuromuscular and biomechanical characteristics refer to the interaction between the neural and musculoskeletal systems and their ability to convert power output into translocation and therefore performance [5]. The multifaceted concept of RE, with multiple types of efficiency (that is, accounting for the work done and energy lost) may be intuitively understood by scientists, practitioners and coaches, nonetheless it has yet to be defined or discussed in great detail in the literature. The heritability of genetic traits is no doubt the prevailing factor affecting RE (Figure 2), however at the moment, there is limited research examining specific genotypes related to better economy [11,12]. Furthermore, many of these factors are modifiable through various training modalities (Figure 2). Therefore RE is an important measure for coaches, athletes and practitioners to understand, quantify and attempt to enhance. The purpose of this review is to 1) examine and review how RE is defined and measured and 2) consider the metabolic, cardiorespiratory, biomechanical, and neuromuscular components that determine RE.

## Defining and measuring running economy

### Defining running economy

Work economy for a given task has emerged as a measurement which is both conceptually clear and practically useful for the evaluation of endurance activities and has become almost universally accepted as the physiological criterion for ‘efficient’ performance [13]. Despite this, there is a discrepancy over the term RE and its definition. Conley and Krahenbuhl (1980) define economy as submaximal oxygen consumption (VO_2submax_) [1]. Williams (1985) refers to VO_2submax_ for a given task as the “physiological efficiency” and Goldspink (1977) claims that economy usually refers to muscle efficiency [7,14]. According to Taylor [15] muscles produce economic force rather than efficient work during running. Efficiency refers to the ratio of work done to energy expended, and thus the terms “efficient” and “efficiency” should *not* be used to relate the energy demands of running to velocity of running because running velocity represent only part of the work being performed by the body while it is transported from one point to another [2]. Other terms such as “cost,” “oxygen cost,” “energy cost,” and “requirement” have all found their way into the literature as ways of describing the relationship between oxygen consumption (VO_2_) and running velocity [2]. The energy cost of running reflects the sum of both aerobic and anaerobic metabolism, and the aerobic demand, measured by the VO_2_ in L^.^min^−1^ at a given speed does not necessarily account for the energy cost of running, which is measured in joules, kilojoules, calories or kilocalories of work done [2,3,16].

Running economy is represented by the energy demand for a given velocity of submaximal running and expressed as the submaximal VO_2_ at a given running velocity [1-3]. This value reflects gross or total economy; a measurement that represents the metabolic, cardiorespiratory, biomechanical and neuromuscular components of running without consideration for what portion of that VO_2_ is a function of good or bad mechanics as opposed to being related to differences in metabolism or force production which may exist in different athletes or under different conditions [2,5,10]. Accordingly, the measure of RE may be flawed as it is determined by multiple variables that may or may not be based on oxygen consumption alone, nevertheless, having an understanding of the underlying idea of RE provides insight into the complexity of this measurement*.* Still being able to describe the VO_2_ related to a particular velocity of running provides a useful way of comparing individuals, or any individual with him or herself under various conditions, and this VO_2_ gives a measure of *running economy*.

### 2.2 Measuring and expressing running economy

The standard approach to quantifying RE involves measuring VO_2_ while running on a treadmill at various constant speeds for a duration long enough to achieve physiological steady-state. Typically, durations of 3 to 15 min have been used in studies if the speed is below the ventilatory/lactate threshold [8], since above this intensity, a slow component of VO_2_ is evident [17]. Often, the steady-state condition is verified by considering other physiological parameters such as verifying that blood lactate concentration are similar to baseline levels [18] and the respiratory exchange ratio (RER) is < 1 [1]. Comparisons between individuals RE are traditionally made by interpolating the VO_2_ to a common running velocity and expressing RE relative to body mass per minute (ml^.^kg^-1.^min^−1^) or by the total volume of oxygen needed to run one kilometer relative to body mass (ml^.^kg^-1.^km^−1^) [19]. The most commonly used reference velocity is 16 km^.^hr^−1^ (268 m^.^min^−1^ = 4.47 m^.^s^−1^), which represents 6 minutes per mile, or 3 min 44 sec per km, however, velocities from 12 to 21 km^.^hr^−1^ appear in the literature [1,3,6,20-32]. In running, however, allometric scaling to the power of 0.67 or 0.75 (e.g. ml^.^kg^-0.67.^min^−1^ or ml^.^kg^-0.75.^min^−1^) has also been reported in order to compare RE between individuals and animals with varying body mass [22,33-47]. However, assessing RE by simply measuring VO_2_ does not take into account differences in substrate use at any given running speed, therefore some studies have expressed RE as the caloric unit cost (kcal^.^kg^-1.^km^−1^) [16,48-51].

### Normative data

**Figure 3:**
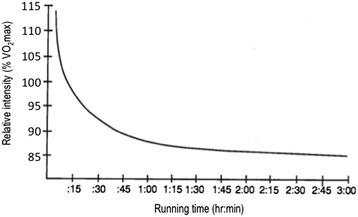
**Relationship between race duration and relative intensity.**

From studies to date it is difficult to accurately ascertain what is good, average, and poor RE due to variation in protocols, gas-analysis equipment, data averaging techniques and differences in maximal aerobic capacity. However, acknowledging these potential limitations, representative VO_2_ values for different caliber of runners from the existing literature are presented in Table 1. The lowest reported value for VO_2_ at 16 km^.^hr^−1^ is 39.0 ml^.^kg^-1.^min^−1^ in an individual East African runner, capable of running 1500 m in 3:35 with a VO_2_max of only 63 ml^.^kg^-1.^min^−1^ [19]. However, the current Men’s Half Marathon World Record holder’s (Tadese Zerisenay, 58 min 23 s; VO_2_max = 83.0 ml^.^kg^-1.^min^−1^) RE was measured at 150 ml^.^kg^-1.^min^−1^ at 19 km^.^hr^−1^ (317 m^.^min^−1^) which is equivalent to 40.0 ml^.^kg^-1.^min^−1^ at 16 km^.^hr^−1^ or 48.2% relative intensity of effort compared to 61.9% of the aforementioned athletes VO_2_max [26]. The concept of relative intensity is an important one because trained runners all perform at near equal percentages of their respective VO_2_max depending on the distance of the event in question (Figure 3) [22,52]. Other examples of exceptional RE include Paula Radcliffe (Women’s Marathon World Record holder, 2 hr 15 min 25 s; VO_2_max = 75.0 ml^.^kg^-1.^min^−1^) 44.0 ml^.^kg^-1.^min^−1^ at 16 km^.^hr^−1^ [53]; Frank Shorter (Men’s Olympic Marathon Gold [1976] and Silver [1980] medalist; VO_2_max = 71.3 ml^.^kg^-1.^min^−1^) 57.0 ml^.^kg^-1.^min^−1^ at 19.3 km^.^hr^−1^ [29]; and Jim Ryun (former Men’s 880 yd,1 min 44.9 s; 1500 m, 3 min 33.1 s; 1 mile, 3 min 51.1 s World Record holder; VO_2_max = 78.3 ml^.^kg^-1.^min^−1^) 48.3 ml^.^kg^-1.^min^−1^ at 16 km^.^hr^−1^ [54].

**Table 1:** **Normative running economy data for male and female runners of varying ability levels**

		**Male mean (range)**		**Female mean (range)**	
**Runner classification**	**Speed (km** ^**.**^ **hr** ^**−1**^ **)**	**Running economy (ml** ^**.**^ **kg** ^**-1.**^ **min** ^**−1**^ **)**	**VO**_**2**_ **max (ml** ^**.**^ **kg** ^**-1.**^ **min** ^**−1**^ **)**	**Running economy (ml** ^**.**^ **kg** ^**-1.**^ **min** ^**−1**^ **)**	**VO**_**2**_ **max (ml** ^**.**^ **kg** ^**-1.**^ **min** ^**−1**^ **)**
Recreational [19,77,202,220-223]	10	36.7 (35.4-38.8)	54.2 (51.0-57.8)	37.7 (32.8-42.6)	49.7 (45.2-54.1)
	12	42.2 (40.4-45.3)		43.2 (38.5-48.1)	
	14	47.4 (46.0-49.5)		47.3 (40.1-51.9)	
Moderately trained [94,224-229]	12	40.7 (37.4-48.1)	62.2 (56.6-69.1)	41.9 (28.9-41.7)	55.8 (50.5-59.4)
	14	46.8 (42.0-55.5)		47.9 (41.3-53.5)	
	16	51.4 (51.6-62.3)		52.9 (45.7-61.0)	
Highly trained [1,21,23,27,31,230,231]	12	n/a	70.8 (65.3-80.2)	41.3 (33.3-50.2)	61.7 (56.2-72.3)
	14	45.0 (32.4-56.5)		48.3 (39.0-56.7)	
	16	50.6 (40.5-66.8)		54.5 (46.2-61.9)	
	18	58.1 (48.0-72.0)		58.6 (54.4-67.1))	
	20	66.5 (65.7-71.6)		n/a	
Elite [21,22,29,31,58,232]	14	39.9 (36.1-44.5)	75.4 (68.2-84.1)	41.9 (38.7-46.9)	66.2 (61.1-74.2)
	16	47.9 (43.2-53.4)		48.9 (45.1-55.8)	
	18	55.9 (50.5-62.3)		56.1 (51.8-63.8)	
	20	63.91 (57.5-71.2)		n/a	

n/a = not applicable.

### Treadmill and overground running

Due to the difficulty of obtaining metabolic data during overground running in the field (i.e. during training and competitions), measurements of RE have typically been made in the laboratory on motorized treadmills during which pulmonary gas-exchange is determined during bouts of constant-speed running and analyzed using various forms of manual (i.e. Douglas bag method) [55] or automated (i.e. breath-by-breath analyzers) gas-analysis systems [56-58]. However, since air and wind resistance are not factors during laboratory testing, transferring treadmill data to overground running requires caution [2,3,8]. Specifically, differences between overground and treadmill running are likely to be found since as speed increases the effects of air and wind resistance become more pronounced (more on air and wind resistance in Kinetics / Ground Reaction Forces section below) [2,59]. Furthermore, the technique of running on a treadmill is different to running over ground where the hamstrings are used to a greater extent to produce propulsive horizontal and vertical forces [60]. For these reasons, data collected during laboratory treadmill testing sessions are typically under-estimations of the true energy demands during over ground running, although a slight incline on the treadmill gradient (~1%) can be used to increase the energy demand in compensation for the lack of air resistance experienced during overground running [60]. In recent years, however, lightweight, accurate, portable telemetric metabolic measuring systems have been designed that enable researchers and practitioners to obtain measurements during running outside of a laboratory environment. However, Saunders et al. [3] caution that careful attention must be made to ensure post- or repeated-measure results are not influenced by changes in environmental conditions.

### Reliability of running economy

In order to evaluate the effectiveness of running training throughout the season or the effect of specific interventions aimed at improving RE, the intraindividual variation (typical error) of RE should be considered. Factors such as treadmill running experience [61], training level [62], footwear [31,61-64], time of day of testing [31,61-64], prior training activity [61,64], nutritional status [62,64], testing equipment [31] and laboratory environment [31] may affect the test-retest reliability of RE measures. Well-controlled studies using moderately trained to elite caliber subjects report intraindividual variation in RE between 1.3% and 5% at speeds between 12 and 18 km^.^hr^−1^ [20,27,31,32,57,61-64], indicating that within-subject results are relatively stable. While no patterns emerge between the training status or gender of the athlete and reliability of RE, it does appear that the typical error is less at running velocities at which the athletes typically train [31,61,62]. While durations of up to 15 min have been used in the assessment of RE [62], multiple ~4 min stages at progressively faster running speeds (e.g. 12, 14, 16, 18 km^.^hr^−1^) have been used in moderate to highly trained runners familiar with treadmill running because steady-state VO_2_ can be reached within 2–3 min at each running speed [31].

Additionally, Hopkins [65] has proposed the concept of the smallest worthwhile change (SWC) to determine the practical significance of interventions. The SWC identifies the magnitude of change required to elicit a meaningful or significant improvement in RE. The SWC, calculated as a proportion of the effect size, represents the magnitude of improvement in a variable as a function of the between-athlete standard deviation of the particular cohort [66,67]. Saunders et al. [31] estimated a SWC of 2.6%, 2.4%, and 2.2% for RE at 14, 16, and 18 km · h^−1^ respectively in 70 highly trained distance runners. Therefore, a distance runner must improve their RE by ~2.2-2.6% before a coach or practitioner can be reasonably confident that a real change (improvement) has occurred. However, the data reported by Saunders et al. [31] is for VO_2_ at a given absolute speed across all subjects. The SWC may be considerably less than this if RE is assessed at the same relative speed and expressed as either an oxygen required to cover a given distance or energy demand (in relation to substrate utilization using RER) to run a given distance [16,68].

## Metabolic and cardiorespiratory efficiency and running economy

In the context of improving RE, metabolic and cardiorespiratory efficiency refers to processes that result in better use of oxygen (increased energy production) relative to a given work output. Fluctuations in cardiorespiratory measures [heart rate (HR), minute ventilation (V_E_)], thermoregulation [core temperature (C_Temp_)], and substrate metabolism [muscle contractile efficiency, mitochondrial efficiency] have been associated with changes in RE [3,69-73].

### Cardiorespiratory measures

Bailey and Pate [69] and Pate et al. [74] have suggested that changes in cardiorespiratory measures (HR and V_E_) are partly responsible for changes in RE during submaximal and maximal exercise. Thomas et al. [75] found a correlation of r = 0.79 (p < 0.05) between changes in V_E_ and changes in oxygen demand during a 5-km race in trained female runners. The increased oxygen demand was postulated to be caused by the increased O_2_ demand of breathing [76]. Franch et al. [77] also reported a correlation (r = 0.77; p < 0.0001) between improvements in RE and reductions in pulmonary ventilation which may account for 25-70% of the decrease in aerobic demand after an intense run training program in recreational runners. Another study attempted to determine the impact of a simulated 5-km race on RE, V_E_, HR, and C_Temp_ [4]. Consistent with other findings [69,75], RE decreased significantly and V_E_, HR, and C_Temp_ all increased significantly from the beginning to the end of the 5-km run. Similar to previous studies [75,77], the increase in V_E_ was the only measure related to the increased RE (r = 0.64; p < 0.05). The fact that the two variables were correlated in several studies does not in itself imply cause and effect; however, quantitative estimates of the reduced cost of breathing with the training-induced decrement in V_E_ suggest that ventilatory adaptation may indeed play a role in improving RE [77].

Interindividual variation in RE has been linked to differences in HR and V_E_. In a report by Pate et al. [74] involving 167 habitual runners, both HR and V_E_ were significantly and positively correlated with VO_2_, indicating that better RE was associated with lower HR and V_E_. Myocardial VO_2_ also constitutes a fraction (1-2%) of whole body VO_2_ during exercise [78]. Reductions in myocardial VO_2_ would result in improved RE from a more efficient combination of HR and stroke volume (i.e. a reduction in HR and increase in stroke volume) [74]. However, according to Bailey and Pate [69] it is unlikely changes in HR make a significant contribution to changes in RE. A 20-bpm change in HR only increased VO_2_ by 8 ml^.^min^−1^, which increased RE from 41.8 to 41.9 ml^.^kg^-1.^min^−1^. Whereas, voluntary hyperpnoea at rest, which increased V_E_ from 70 to 100 ml^.^min^−1^, has been found to increase VO_2_ by 122 ml^.^min^−1^ [79]. If training is able to decrease the work of breathing at a specific running velocity, this could contribute to an improved RE [69]. Using recent cost estimates of exercise V_E_, the cost of V_E_ increased O_2_ consumption by 31–50 ml (0.4-0.6 ml^.^kg^-1.^min^−1^) in men and 19–31 ml (0.3-0.5 ml^.^kg^-1.^min^−1^) in women. This explains 12-19% of the increase in VO_2_ in men and 16-26% for the women [80,81]. Other estimates have found the work of ventilation to constitute up to 6-7% of the total oxygen cost of exercise [82]. Thus variables other than V_E_ are also responsible for the changes in RE.

### Body temperature

There is conflicting evidence regarding the relationship between C_Temp_ and RE [63,69]. In some studies, a higher C_Temp_ has resulted in an increase in VO_2_ at a given speed under hyperthermic conditions [83,84], likely due to increases in metabolic demand from augmented circulation, sweating, V_E_, and a decrease in efficiency of oxidative phosphorylation [84-86]. In this regard, Grimby [87] found that a 1.3 ° C increase in C_Temp_ increased VO_2_ by 5.5% and Thomas [4] found a slightly greater change in VO_2_ (6.2%) after a 1.0 ° C increase in C_Temp_. In contrast, results from other studies [88,89] indicate no change or a reduction in VO_2_ occurred during hyperthermic exercise, suggesting a higher C_Temp_ enhanced mechanical efficiency of muscle to a degree equal to or greater than the increase caused by changes in circulation, sweating, and V_E_.

### Muscle fiber type

It is now accepted that a range of muscle fiber types exist in humans [90,91] and that each exhibit their own metabolic characteristics [71]. Indeed, the structure and composition of muscle fibers seems to influence RE [71,92,93]. Type IIA fibers are more oxidative than type IIX fibers and have functional characteristics more similar to type I fibers [94]. Type-II specific myosin ATPase isoforms require 1.6- to 2.1-fold more ATP per unit force production than type I and therefore require a proportionately higher oxidative phosphorylation [95]. Therefore an increase in type IIA fibers should increase the oxidative capacity of muscle and should contribute to improved RE. Although no research has examined the genetic link between muscle fiber type and RE, athletes may be predisposed to better or worse economy based on their composition of type I and type II muscle fibers [96]. Current results suggest there are mixed findings between muscle fiber type and RE [90-93,97].

## Biomechanics and running economy

Running involves the conversion of muscular forces translocated through complex movement patters that utilize all the major muscles and joints in the body [5]. Current evidence suggests that a variety of biomechanical characteristics are likely to contribute to RE; these include a variety of anthropometric dimensions [73,91,98-103], select gait patterns [104-111], and kinematic and kinetic factors [46,91,103,111-117] that have been shown to affect biomechanical efficiency and relate to better RE.

### Anthropometric characteristics

#### Body mass and mass distribution

A variety of anthropometric characteristics such as height, body mass, physique, and segmental mass distribution may help explain interindividual and group differences as well as potential influences on RE. It is well-known that the oxygen demand of running does not increase proportional to body mass [15,101], and the VO_2_ per kilogram of body mass is higher in children than adults [2,8,118-128]. Indeed, in running events ranging from 800-m to the marathon, it’s not uncommon to see individuals range by as much as 25 kg and/or 30 cm in the same race, even at the elite level. Several studies [129,130] have shown lightweight men to be no more or less economical than their heavier counterparts. Other research has demonstrated that when body mass is artificially increased by adding weight to the trunk during running, VO_2_ per kilogram of body mass decreases both in children [128,131,132] and adults [128,131,133]. Consequently, several authors have suggested that the lower submaximal VO_2_ in adults compared with children is a function of differences in body mass and not merely growth and maturation [39,134]. In support, several studies [73,91,102,103] have shown small to moderate inverse relationship between body mass and RE. Arellano and Kram [45] suggest body weight support and forward propulsion comprises ∼ 80% of the net metabolic demand of running, while the task of leg-swing comprises ∼ 7%, maintaining lateral balance ~2% and arm-swing actually reduces the demand by ∼ 3%, indicating a net metabolic benefit.

The relationship between body mass and RE has been proposed to be a result of individual differences in mass distribution within the body, particularly in the limb segments [98]. For example, subtle differences in physique, particularly a low body mass index and long slender legs where the majority of mass is distributed higher on the thigh, have been suggested to be the primary reason for the extraordinary RE of African runners [19,25,135,136]. Although it is difficult to obtain a direct measure of the relationship between segmental mass distribution and RE, indirect support comes from experimental studies in which mass has been added to the lower limb segments of runners [137-143]. In general, the results from these studies indicate that the aerobic demand of carrying an extra load becomes more significant when the mass is located more distally. Myers [141] found that the aerobic demand of carrying an extra kilogram on the trunk is increased by 1% whereas when an equal mass is carried in the shoes, aerobic demand is increased by 10%. Other studies have found an increased VO_2_ of 4.5% [144] and 14% [140] per kilogram carried on the feet and 7% increase when carried on the thigh [140]. Given the distal location of the feet, foot size or foot size relative to body size would also suggest it influences RE [5]. If one considers that a typical standard shoe weighs about 350 g, about 200 g more than most minimal shoes, and that the aerobic demand for every 100 g added to the trunk increases by about 0.1% and added to the foot increases by 1% [141,145], then the results from Perl et al. [146] suggest the net savings to minimal-shoe running is between 4.4% and 6.8%. These results support previous studies [142,143,147-153] reporting running barefoot or in minimal shoes to be more economical than running in standard shoes. However, cushioning and other features of shoe design besides weight have been shown to have significant effect on RE [145]. For example, the aerobic demand during treadmill running was about 2.8% less when running in well cushioned shoes compared with poorly cushioned shoes of similar mass [145].

#### Limb length

While lower limb mass distribution has been shown to affect RE, there is no consensus on whether leg length is a factor in determining RE. Humans and animals of different sizes use approximately same amount of energy to run {Roberts, 1998 #1329;Roberts, 1998 #820}. Running involves little work against the environment; work is done by muscles and tendons to lift and accelerate the body and limbs. Some of the work is recovered from muscle-tendon springs without metabolic cost, however, regardless of the amount of work muscles do, the limbs must be activated and develop force to support the weight of the body {Kram, 1990 #821}. Leg length contributes to angular inertia and the metabolic cost of moving legs during running [5,154], and while there has been some research focusing on the relationship between leg length and stride length [99,155], the influence of leg length on economy has only been investigated indirectly. Research examining the physiques of male and female sprinters, middle-distance and long-distance runners have characterized sprinters as short-legged and middle- and long-distance runners and long-legged [156]. In general middle- and long-distance runners have been found to exhibit better economy than sprinters [22,29,92,102], however the influence of leg length on these differences is unknown. Myers and Steudel [141] suggest that for a given body mass, speed and gait pattern, runners that are smaller and have proportionately greater amount of body mass distributed proximally in the legs perform less work to accelerate and decelerate the limbs. However, despite Williams and Cavanagh [91] finding a large variation in RE among 31 male distance runners, there were no differences associated with segmental leg lengths and masses.

#### Achilles tendon moment arm

**Figure 4:**
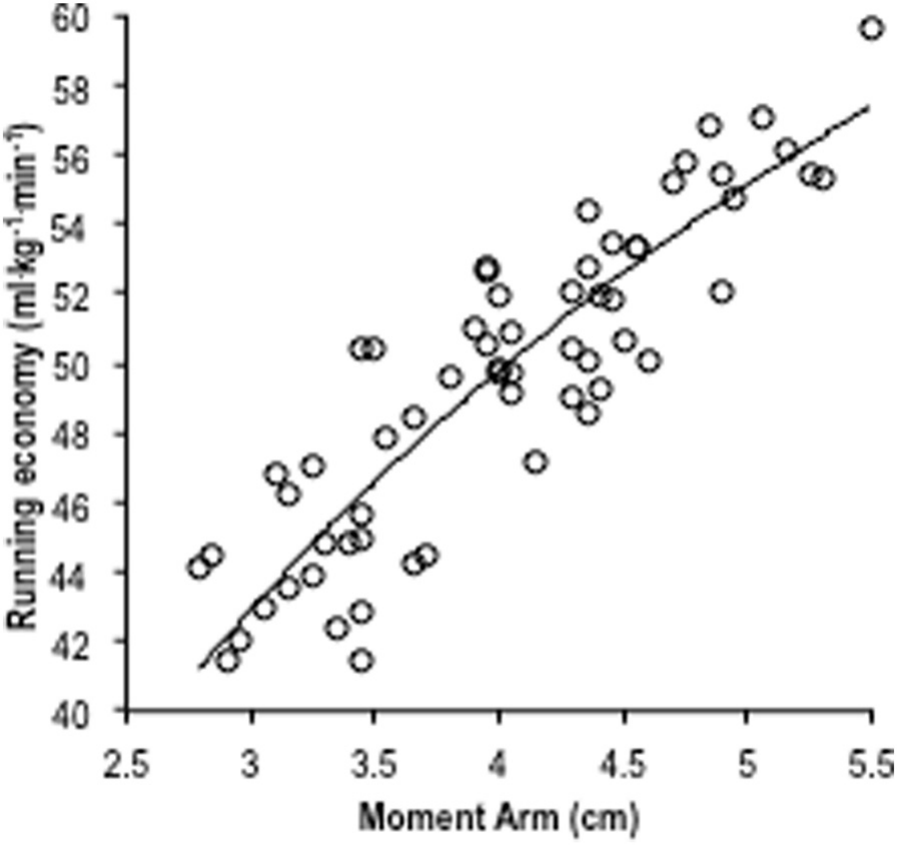
**Relationship between moment arm length and running economy at 16 km** ^**.**^ **hr** ^**−1**^ **and moment arm (r = 0.90).** Reproduced from Barnes et al. {Barnes, 2014 #1278} with permission.

The amount of energy stored in a tendon depends on the mechanical properties of the tendon and on the forces that stretch the tendon, such as foot length. Kinetic and potential energy removed from the body in the first half of the stance phase is stored briefly as elastic strain energy and then returned in the second half by elastic recoil [157]. Thus, for a given kinematic pattern, and hence kinetic pattern, tendon force is inversely related to the moment arm of the Achilles tendon [100]. Since it is generally accepted that storage and reutilization of elastic energy in tendons substantially reduces energy demands in running [158] previous research has been able to establish a moderate [159], large [100] and extremely large {Barnes, 2014 #1278} (Figure 4) relationship between the variation in RE and the moment arm of the Achilles tendon, albeit in small sample sizes of 8 to 63 {Barnes, 2014 #1278;Raichlen, 2011 #606;Scholz, 2008 #605}. Shorter Achilles tendon moment arm length and less flexible lower limb joints are associated with improved RE [100,160,161].

Other anthropometric characteristics throughout the body have also been investigated. Foot length has been found to be negatively correlated with RE in elite male runners [103]. Pelvic and shoulder width could theoretically have an influence on RE [5] but have been studied very little with available evidence suggesting either no relationship [91,112], or a moderate negative correlation between pelvic width and RE [103]. The only postural characteristic that has been investigated relative to RE is trunk angle or degree of forward lean while running. When comparing distance runners grouped by RE, Williams and Cavanagh [91] found that the most economical group displayed a slightly greater forward lean (5.9°) compared with the middle (3.3°) and least (2.4°) economical groups.

### Running style / gait patterns

There is a belief amongst practitioners that, over time, runners adopt their most economical running style [162,163]. Accordingly, high training volumes and the number of years of running experience have been suggested to be important for improved RE [164]. Indeed, a number of studies show that individuals tend to freely choose their most economical gait pattern [13,104,165-167]. While studies have identified small to moderate relationships between biomechanical characteristics and RE [91,104,111,166,168-170], stride length is one of the few gait variables that has been shown by direct experimental evidence to affect economy [104-106,171].

#### Stride length and stride rate

Results from a number of studies [104,166,167,171,172] have indicated that submaximal VO_2_ increases curvilinearly as stride length is either lengthened or shortened from that self-selected by the runner. This basic curvilinear relationship between stride length and economy has also been shown for walking [173] and racewalking [106]. The basic assumption behind this research appears to be that strides which are too long will require considerable power during propulsion, excessive vertical oscillation of the center of mass, produce a foot strike position which creates large braking forces and require joint ranges of motion which invoke increased internal friction and stiffness [5]. Conversely, strides that are too short would increase internal work of contracting muscles through increased frequency and reciprocal movements, resulting in increased energy expenditure and decrease in RE [5].

Previous research has shown that VO_2_ was lowest at stride lengths close to the self-selected condition [104,171]. Based on these results, Cavanagh and Williams [104] concluded that there is little need to dictate stride length for most runners since they already tend to display near optimal stride lengths. They proposed two mechanisms for this phenomenon. First, runners naturally acquire an optimal stride length and stride rate over time, based on perceived exertion [91], which supports the premises put forth previously [91,162,164]. Second, runners may adapt physiologically through repeated training at a particular stride length/stride rate for a given running speed [104].

Kaneko et al. [166] suggested that the link between stride rate and economy may be associated with muscle fiber recruitment. At slower stride rates (and longer stride lengths), the muscles need to develop relatively high external power during propulsion to overcome large braking forces. Conversely at fast strides rates (short stride lengths), the mechanical power associated with moving the limbs increases due to increased frequency of reciprocal movements. They indicated that these extreme conditions may require a greater reliance on less economical Type II fibers than more intermediate stride rate/stride length combinations [166]. Consequently, efforts to improve RE via stride rate manipulation would be ineffective, unless the runner’s freely chosen stride rate is not economically optimal [69]. Kram and Taylor {Kram, 1990 #821} suggest differences in oxygen demand are proportional to stride rate at equivalent speeds, suggesting that the time available for developing force is important in determining RE.

#### Vertical oscillation

Studies comparing the biomechanical characteristics of elite and good runners, found that elite distance runners have slightly less vertical oscillation and had better RE than good runners [107,110,111,114]. Similarly, Williams and Cavanagh [91] showed a trend, although nonsignificant, towards less vertical oscillation and better RE. The intuitive perception is that vertical oscillation is adversely related to economy; however, Cavagna et al. [107] reported that less vertical oscillation results in high stride frequency and higher internal work to accelerate lower limb segments, thus increasing oxygen demand and reducing RE. Conversely, Halvorsen et al. [174] showed that reducing vertical oscillation has a positive effect on RE.

#### Footstrike patterns

It continues to be argued that a forefoot strike pattern during running is more economical than a rearfoot pattern; however, previous studies using one habitual footstrike group have found no difference in RE between footstrike patterns [103,108,109]. In fact, Gruber et al. [175] found no differences in VO_2_ between 19 habitual forefoot runners and 18 habitual rearfoot runners. However, when subjects ran with the alternative footstrike pattern, VO_2_ increased significantly (5.5%, p < 0.001) with the forefoot pattern but not the rearfoot pattern. Contrary to popular belief, these results suggest that the forefoot pattern is not more economical than the rearfoot pattern.

### Kinematics and kinetics

While stride length, stride rate and other gait related characteristics have been associated with RE, other kinematic and kinetic factors such as angular velocities of limb segments and joints [91,103,111-114] and ground reaction forces [46,115-117] have also demonstrated a relationship with RE.

#### Lower body kinematics

Comparisons of elite and good distance runners indicate that better economy in elite runners was associated with greater maximal angle of the thigh during hip extension, more extended lower leg at foot strike, more acute knee angles during swing and toe-off and that good runners plantar flexed an average of 10° more during toe-off than elite runners [103,113,114]. Whereas running with experimentally increased knee flexion (‘Groucho running’) has been shown to increase the oxygen demand of running by as much as 50% [176]. Results from a number of studies indicate relationships between economy and joint angles and velocities were trivial to moderate in strength [91,103,113,114,177,178], suggesting optimal kinematic patterns may be specific to the individual.

#### Upper body kinematics

Most investigations of RE and running mechanics have focused on the kinematics of lower limbs with only a few studies [103,111,112] considering the upper body limbs. Anderson and Tseh [112] found no relationship between RE and should width, hip width, or ratio of shoulder:hip width. Whereas Williams and Cavanagh [103] found a moderate negative correlation between shoulder:pelvic width and RE in elite male runners, indicating that the moments and forces generated by counter-rotations of the shoulders and hips and movements of the arms may effect RE [179-181]. Accordingly, a positive correlation between economy and angular velocity of shoulder rotation and angular displacement of the hips and shoulders about the polar axis of the trunk [112] as well as a negative correlation between economy and angular displacement of the shoulder in the sagittal plane has previously been described [112]. However, Tartaruga et al. [111] found no relationship between velocity changes of the wrists and shoulders or rotation of the hips and shoulders relative to the polar axis of the trunk and economy. Some results have shown less arm movement, as measured by wrist excursion during the gait cycle, tended to reduce total upper body excursion from the body center of mass both laterally and horizontally and be associated with better RE [91,111,112,181] .

#### Kinetics / ground reaction forces

Investigations related to kinetics and RE are limited and most work has focused on vertical ground reaction forces. Kram and Taylor [46] presented a simple inverse relationship between the aerobic demand during running and the time the foot applies force to the ground during each stride, independent of body mass, indicating that the energy demand during running is determined by the cost of supporting one’s body mass and the time course of generating force. Williams and Cavanagh [91] surmised that more economical runners have identifiable kinetic patterns in their running style, which are recognizable by less wasteful vertical motion [117]. However, data collected from elite male and female distance runners showed relatively low correlations between ground reaction forces and RE [103,113] suggesting that ground reaction forces are not likely to be the determining factor that makes one runner more economical than another, and that in fact some elite runners are economical despite low ground reaction forces.

While vertical ground reaction forces have been shown to affect the metabolic demand during running in recreational and moderately trained runners due to the requirement to support body mass [46,115-117], horizontal forces can also substantially affect RE. For example, in 25 well-trained endurance athletes, Nuumela et al. [168] reported mass-specific horizontal forces were substantially related to RE at five different running speeds. Similarly, Storen et al. [182] found that the sum of horizontal and vertical peak forces were inversely correlated with RE (r = 0.66).

## Neuromuscular characteristics and running economy

In addition to metabolic, cardiorespiratory, and biomechanical factors, neuromuscular characteristics are also important aspects of RE. The interaction between the neural and muscle systems (i.e. neuromuscular system) is fundamental to all movement, and effectively translates cardiorespiratory capacity into efficient mechanics and therefore into performance. It is becoming more evident that aerobic factors are not the only variables that affect endurance performance [10]. In fact, Green and Patla [183] suggest that any failure of the contractile machinery could prevent full utilization of available oxygen, suggesting that in some cases, the ability to use available oxygen might not be the limiting factor in endurance performance. Essentially neuromuscular efficiency can be divided into two categories: 1) factors that improve the neural signaling and motor programming of the running motion and 2) those that improve the muscle force production itself.

### Neural signaling and motor programing

High performance running is a skill, much like hitting a golf ball or shooting a basketball, that requires precise timing of nearly all the major muscles and joints in the body to convert muscular force in translocation [5]. Similar to those skills, practice is needed to improve the efficiency at the activity. Motor learning studies have shown that continued practice of a task results in more skilled control of movement, characterized by decreased amplitude and duration of muscle activity, decreased muscle co-activation and less variability of movement [97,184,185]. Recent evidence has shown that recreational runners (3.4 ± 2.8 km^.^wk^−1^) exhibited greater individual variance (i.e. variability between strides), greater population variance (i.e. variability of muscle recruitment between athletes), more extensive and more variable muscle co-activation and longer durations of muscle activity than moderately trained runners (6.6 ± 1.4 years of running experience, who ran 61.4 ± 8.8 km^.^wk^−1^) [186]. These findings are consistent with previous short-term training studies of arm, hand and leg (pedaling) movements [185,187-189], suggesting that ongoing neuromuscular adaptations occur as a result of continued training. It is apparent within the literature that run training can induce positive changes in RE [118,190,191]. Running appears to induce adaptations in motor programing and recruitment that are critical for superior RE [5,184,185]. If neuromuscular adaptations are responsible for the changes in RE then it would be reasonable to suggest that there would be alterations in neural signaling during running following training. Bonacci et al. [10] advocate that adaptations to motor recruitment as a result of training represent a learning effect. Positive adaptations infer that an individual learns to produce specific patterns of muscle recruitment that are associated with improved efficiency of the task (e.g. improved biomechanical and neuromuscular efficiency) resulting in enhanced performance [10].

### Muscle force production and stiffness

Their are two muscle contraction-related issues that potentially influence energy demand and RE; velocity of contraction and balance between concentric and eccentric contractions. With regards to velocity of contraction, Taylor [15] has observed that it is less costly for muscles to generate force at low velocities, that force is highest and metabolic rate lowest during isometric contraction, and that the energy cost of generating force increases dramatically with great shorting velocity. The proposed mechanism for this is that muscle contractions are primarily isometric, adjusting the stiffness of the muscle-tendon unit during the eccentric phase to produce simultaneous deceleration and elastic stretch, then producing a nearly isometric impulse that initiates ballistic concentric acceleration. This proposed mechanism would promote optimization by exploitation ‘free’ elastic energy, and minimizing metabolic requirements. Such optimization would obviously demand precise timing, and integration and refinement of the temporal, kinetic and kinematic patterns, which would require considerable practice and training.

### Muscle power

It has been suggested that endurance performance may be limited not only by aerobic power but also by ‘muscle power’ factors related to the force and velocity characteristics of the neuromuscular system [192]. Indeed, performance during a 5-km and 10-km run has been shown to be partially determined by neuromuscular characteristics and muscle power, suggesting the skeletal muscle contractility differs between fast and slow runners [192-194]. Similarly, in a homogenous group of highly trained endurance runners with similar VO_2_max values, those athletes with faster 10-km and 5-km run times displayed higher relative muscle pre-activation (prior to touchdown), accompanied with lower relative integrated electromyographic (iEMG) activity during the propulsion phase, along with shorter stance phase contact times than those athletes with slower run times [194,195]. Furthermore, there was a significant correlation between RE and mean stance phase contact times during constant velocity running, suggesting muscle power characteristics play an important role in determining distance running performance in highly trained runners [194].

### Lower-leg stiffness

It is possible that shorter stance phase contact times and greater muscle pre-activation may represent enhanced leg muscle stiffness, leading to faster transition from the braking to propulsive phase of ground contact [195,196]. Dalleau et al. [197] highlighted the importance of neuromuscular factors by demonstrating that RE was related to the stiffness of the propulsive leg, with greater stiffness eliciting the best RE. Arampatzis et al. [198] corroborate this finding such that in a group of 28 long-distance runners separated into three groups by economy, the most economical runners had highest tendon stiffness. Leg stiffness is modulated by neuromuscular activation, and changes in stiffness have been shown to occur as a result of neuromuscular adaptation to training [199]. In support of the association between motor recruitment and leg stiffness, a reduction in EMG pre-activation was shown to be significantly related to a decrease in post-landing leg stiffness following fatiguing exercise [200]. Greater duration of muscle co-activation of bi-articular leg muscles during stance has also been significantly associated with better RE [201]. Muscle co-activation modulates leg stiffness during running and may alter RE through utilization of stored elastic energy, which has no additional metabolic cost. Albracht and Arampatzis [202] indicated that increased tendon stiffness is indicative of greater energy storage and return and a redistribution of muscular output within the lower extremities while running, which might result in improved RE. Running economy and stiffness have been shown to change together with training [48,203]. It has also been shown that stiffness of the muscle-tendon unit increases with running speed [105,158,204].

### Stretch shortening cycle

Kyrolainen et al. [177] found that as running speed increased so did EMG preactivation and ground reaction forces, along with their rate of force production. Preparatory muscle function is an important function of the stretch shortening cycle (SSC). The SCC is a combination of a high velocity eccentric contraction followed immediately with a concentric contraction. Stretch shortening cycle muscle function enhances performance during the final phase (concentric action) [205], and the increase in preparatory muscle activity with higher running speeds was suggested to be a mechanism to tolerate higher impact loads, regulate landing stiffness [206] and improve RE [177]. A recent study showed that a greater ratio of eccentric to concentric vastus lateralis muscle activity was associated with a lower metabolic demand during running (i.e. better RE) [207].

### Elastic energy storage

The balance between eccentric and concentric contractions could potentially influence RE, since the eccentric contractions during which elastic energy is stored are less costly than the concentric contractions in which the energy is released [14]. There is clear evidence that the mechanical efficiency of running exceeds the efficiency of conversion of chemical energy to kinetic energy by muscles [13,14,158]. Elastic energy stored during the eccentric contractions of running makes a substantial contribution to propulsion as it is released during subsequent concentric contractions [158,208]. Unfortunately, there currently are no data available from which to quantify the relative energy cost of the two types of contractions nor has there been a method devised to differentiate true eccentric contractions from tendon stretching or to quantify the storage and release of elastic energy [13,14,209-212]. There is however, consensus that this phenomenon contributes to both efficiency and economy of movement.

Both actomyosin cross-bridges and tendons have been implicated as important sites of energy storage [157,213]. Ker et al. [157] have estimated that the Achilles tendon and tendons in the arch of the foot can store 35% and 17%, respectively, of the kinetic and potential energy gained and lost in a step while running at moderate speed. Alexander [214] has shown that in a 70 kg human running at ~16 km^.^hr^−1^, more than half of the elastic energy can be stored in just two springs, the Achilles tendon and the arch of the foot. Cavagna et al. [215] have estimated that VO_2_ during running might be 30 to 40% higher without contributions from elastic storage and return of energy. At higher running speeds, elastic recovery of energy prevails over the contractile machinery and accounts for most of the work [15,158]. The available evidence indicates that there may be substantial interindividual differences in ability to store and release elastic energy [216-218] and it has been suggested that fiber composition, gender and maturity are likely contributors to these differences [219].

## Conclusions

Running economy is a complex, multifactorial concept that represents the sum of metabolic, cardiorespiratory, biomechanical and neuromuscular efficiency during running. It is possible to obtain reliable measures of RE in the laboratory, and a range of values from varying standards of runners is retrievable from current literature. Metabolic and cardiorespiratory factors that affect RE include HR, V_E_, C_Temp_, and muscle fiber type. While, there does not appear to be any easily identifiable or universally applicable biomechanical pattern of ‘efficient’ movement that will apply to all runners, it does appear that runners with a variety of anthropometric characteristics such as a mass distribution closer to the torso and shorter Achilles moment arms tend to have better RE. Neuromuscular efficiency may play an important role in determining RE, especially in athletes with similar physiological attributes. Specifically, the timing and amplitude of muscle activity prior to and in the initial phase of ground contact affect economy by augmenting leg stiffness and the exploitation of stored elastic energy. It is likely that adjusting a given determining factor may result in an economy enhancement in one athlete but the same adjustment in another might be uneconomical because of differences in other physiological or biomechanical characteristics. Perhaps a more promising avenue of research may be to concentrate on the individual runner in an effort to best identify how that athletes structure and functional abilities influence RE, subsequent performance as well as injury susceptibility.
